# Modeling resilient modulus of subgrade soils using LSSVM optimized with swarm intelligence algorithms

**DOI:** 10.1038/s41598-022-17429-z

**Published:** 2022-08-24

**Authors:** Abdelhalim Azam, Abidhan Bardhan, Mosbeh R. Kaloop, Pijush Samui, Fayez Alanazi, Majed Alzara, Ahmed M. Yosri

**Affiliations:** 1 Department of Civil Engineering, College of Engineering, Jouf University, Sakaka, 2014 Aljouf Saudi Arabia; 2 Civil Engineering Department, National Institute of Technology Patna, Patna, India; 3 Department of Civil and Public Works Engineering, Mansoura University, Mansoura, 35516 Egypt

**Keywords:** Engineering, Materials science, Mathematics and computing

## Abstract

Resilient modulus (Mr) of subgrade soils is one of the crucial inputs in pavement structural design methods. However, the spatial variability of soil properties and the nature of test protocols, the laboratory determination of Mr has become inexpedient. This paper aims to design an accurate soft computing technique for the prediction of Mr of subgrade soils using the hybrid least square support vector machine (LSSVM) approaches. Six swarm intelligence algorithms, namely particle swarm optimization (PSO), grey wolf optimizer (GWO), symbiotic organisms search (SOS), salp swarm algorithm (SSA), slime mould algorithm (SMA), and Harris hawks optimization (HHO) have been applied and compared to optimize the LSSVM parameters. For this purpose, a literature dataset (891 datasets) of different types of soils has been used to design and evaluate the proposed models. The input variables in all of the proposed models included confining stress, deviator stress, unconfined compressive strength, degree of soil saturation, soil moisture content, optimum moisture content, plasticity index, liquid limit, and percent of soil particles (P #200). The accuracy of the proposed models was assessed by comparing the predicted with the observed of Mr values with respect to different statistical analyses, i.e., root means square error (RMSE) and determination coefficient (R^2^). For modeling the Mr of subgrade soils, percent passing No. 200 sieve, optimum moisture content, and unconfined compressive strength were found to be the most significant variables. It is observed that the performance of LSSVM-GWO, LSSVM-SOS, and LSSVM-SSA outperforms other models in predicting accurate values of Mr. The (RMSE and R^2^) of the LSSVM-GWO, LSSVM-SSA, and LSSVM-SOS are (6.79 MPa and 0.940), (6.78 MPa and 0.940), and (6.72 MPa and 0.942), respectively, and hence, LSSVM-SOS can be used for high estimating accuracy of Mr of subgrade soils.

## Introduction

Structural responses of pavement are crucial requirements to assess the quality of pavement construction materials under traffic loading changes^1,2^. Thus, based on the Association of State Highway Officials (AASHTO), the pavement characteristics should be qualified in the road design and used^2,3^. Resilient modulus (Mr) of pavement materials is one of the important characteristics, which is defined as the ratio of dynamic deviatoric stress to the recoverable strain under acyclic pulse load^2,4^. For subgrade soil and unbound granular materials, Mr is used to measure the elastic modulus of soil layers at a given stress level, and describe the non-linear stress–strain of soils under dynamic loads^2^. Many studies were devoted to investigating the effect of different factors on the Mr of subgrade soils. These studies concluded that stress state, dry density, aggregate gradation, amount of fines (materials passing the standard US sieve No. 200), moisture content/matric suction, particle shape, and aggregate type have a significant impact on the Mr^3,5–9^.

Normally, Mr determination uses expensive experimental testing and is time-consuming; furthermore, the spatial variability of soil properties and the nature of the test protocols have made Mr's determination complex and inexpedient. Therefore, many researchers utilized the machine learning technique to estimate the Mr of pavement materials^3,4,10–13^. Predicting the Mr of subgrade soils is important in building a relationship between input and output variables for modeling those values. Consequently, the accurate modeling of Mr can help in defining the important variables that can be measured to determine the Mr; which can improve the quality of pavement construction with low-cost measurements.

Recently, hybrid machine learning algorithms, as well as hybrid artificial intelligence, are widely applied to improve the accuracy of conventional approaches^10,13–19^. Integrated support vector machine (SVM) models were used to predict and classify the pavement cracks, and the performance of the integrated was shown to be high^15^. Moreover, an integrated SVM technique was applied in modeling the asphalt pavement performance, and the designed model has accuracy in modeling non-linear pavement behaviors^20^. Also, the SVM was optimized by a filter to design an integrated model for predicting the remaining service life of pavement, and the results showed the correlation index of the proposed model was high (95%)^21^. Herein, SVM and least square support vector machine (LSSVM) are widely used in modeling different pavement characteristics^11,15,22,23^. The LSSVM was used in modeling Mr; however, the accuracy of the conventional LSSVM method is still limited^11,24^.

It is pertinent to mention that LSSVM is a regression-based machine learning model and provides a higher degree of accuracy compared to other conventional machine learning models such as ANN^25^. The outcomes of conventional machine learning algorithms, such as ANN, ELM, etc., are stochastic in nature, and generating the same results over multiple runs is not possible through such algorithms^26^. On the other hand, LSSVM has two hyper-parameters, namely $\gamma$ (regularization parameter) and $\sigma$ (kernel parameter), and for a given value of $\gamma$ and $\sigma$, the LSSVM produces the same results over multiple runs. However, choosing the best values for these parameters for an effective LSSVM model is not only time-consuming, but also yields incorrect results in many circumstances. Generally, researchers use trial-and-error approaches to determine the optimum values of hyper-parameters in predicting the desired output^27–31^. Therefore, this study aims to develop a high-performance hybrid machine learning model for modeling the Mr using LSSVM. Although the hybrid LSSVM models outperform single LSSVM and SVM, the method in modeling nonlinear problems^32^, its use in modeling Mr is still limited. Hybrid LSSVM was applied in different engineering applications, and that performance is shown to be high^24,32–34^. For instance, LSSVM- particle swarm optimization (PSO) was proposed to model slope stability, and the results showed that the performance of the model was high^35^. The errors indicator of LSSVM- symbiotic organisms search (SOS) in modeling pavement rutting distress was found to be small^23^; this means that this model accuracy may be high in modeling other pavement characteristics. LSSVM- grey wolf optimizer (GWO) model was proposed in a non-linear modeling, and the results show the model performance is better than PSO-based predictive models^36^. Salp swarm algorithm (SSA) was integrated with LSSVM, and there are advantages in avoiding the overestimated fitting^37^. For this, swarm optimized algorithms are integrated with LSSVM to model the Mr of subgrade soils in the current study. PSO, SOS, GWO, SSA, slime mould algorithm (SMA), and Harris hawks optimization (HHO) algorithms are used to optimize the LSSVM hyper-parameters and compared to propose an accurate model that can be used in Mr modeling. SMA and HHO are new optimization algorithms (OAs) and those performances were evaluated and assessed^38,39^.

This study aims to: (a) design a novel hybrid LSSVM approaches for modeling the Mr of subgrade soils. (b) develop and compare six models, LSSVM-PSO, LSSVM-GWO, LSSVM-SSA, LSSVM-SOS, LSSVM-SMA, and LSSVM-HHO in modeling the Mr values. (c) evaluate the significance of different input variables in modeling Mr. To address the significance of the variables on Mr modeling, three different soils characteristics [these data were collected by the Ohio Department of Transportation (ODOT)^3^] have been used in the current paper. Confining stress, deviator stress, unconfined compressive strength, degree of soil saturation, soil moisture content, plasticity index, percent of soil particles passing through a #200 sieve, liquid limit, optimum moisture content were used to model the Mr.

## Least square support vector machine (LSSVM)

In order to improve the performance of SVM, the LSSVM was proposed by Suykens and Vandewalle^40^. LSSVM methods work out linear matrix problems with fewer constraint conditions^41,42^. The main advantages of LSSVM are that it overcomes the SVM drawbacks in computation cost and uncertainties in structural parameter determination^41^. Compared to SVM, LSSVM is a more powerful computation in solving nonlinear and small-data problems^43^. LSSVM is used for classification and regression problems. This study aims to develop a high-performance hybrid machine learning model using a regression-based machine learning model, i.e., LSSVM. The regression modeling of LSSVM can be summarized as follows^35,36^:

For training of given points *l*, $\left\{\left({x}_{i},{y}_{i}\right)|i=\mathrm{1,2},3,\dots l\right\}, {x}_{i}\in {R}^{n}$ is input variables and $y\in R$ is the output variable; the regression fitting output of LSSVM can be expressed as:

1 $$y\left({x}_{i}\right)={w}^{T}\varphi \left({x}_{i}\right)+b$$

Based-optimizing formulas:

2 $${\mathrm{min}}_{w,\beta }J\left(w,\beta \right)=0.5{w}^{T}w+\frac{c}{2}\sum_{i=1}^{l}{\beta }^{2}$$

3 $${\mathrm{s}.\mathrm{t}. \, y}_{i}\left({x}_{i}\right)={w}^{T}\varphi \left({x}_{I}\right)+b+\beta, \,\,\,\, i=\mathrm{1,2},\dots,l$$

where, $w, b \, {\text{and}} \, \beta$ are weight vector, deviation, and error variation, respectively; $\varphi \left(.\right)$ denotes the mapping function, and $c \in {R}^{+}$ is penalty parameter. Largrange method is utilized to solve the above equations^40^.

The linear transformation is applied to solve the Lagrange parameters as follows^36^:

4 $$\left[\begin{array}{cc}0& {1}_{l}^{T}\\ {1}_{l}& K+{c}^{-1}{I}_{l}\end{array}\right]\left[\begin{array}{l}b\\ \alpha \end{array}\right]=\left[\begin{array}{l}0\\ y\end{array}\right]$$

where, $\alpha$ is a Lagrange multiplier, K is the kernel function ($K={\varphi \left({x}_{i}\right)}^{T}\varphi \left({x}_{j}\right), \left(i,j\right)=\mathrm{1,2},\dots,l$).

In this study, the radial-basis function is selected, which was performed in similar studies and found to be the best in modeling non-linear behaviors^11,35^, and it can be expressed as follows^36^:

5 $$K=K\left(x,{x}_{i}\right)={exp}^{\frac{{-|x-{x}_{i}|}^{2}}{2{\sigma }^{2}}}$$

The $\sigma$ is the kernel function width.

Therefore, the fitting model of the final output can be expressed as:

6 $$y\left(x\right)=\sum_{i=1}^{l}{\alpha }_{i}K\left(x,{x}_{i}\right)+b$$

Here, the main disadvantage of LSSVM is that accuracy depends on the regularization parameter ($\gamma$) and kernel function parameter, i.e., kernel width ($\sigma$) to improve the modeling results. Although the effectiveness of reconstructed input datasets with optimal parameters of conventional LSSVM in some cases^43^, it may have some inherent bias with changing the trend of other cases. In addition, time consuming and a priori knowledge requirements may decrease the model accuracy. For that swarm, algorithms have been developed to provide effective LSSVM parameters that reduce the bias of changing the data inputs to overcome the time consuming and a priori information requirements. This study uses the following swarm intelligence meta-heuristics algorithms to optimize those parameters. Herein, it should be mentioned that the proposed model can be used to optimize Mr within the limitation of input datasets.

## Swarm intelligence meta-heuristic algorithms

### Particle swarm optimization (PSO)

PSO is a population solution-based social search behaviour of swarm members “particles”^35^. It begins with random initialization of particles in the search space to build their own and neighbours’ previous successful attempts^35,38^. This aims to find the best position of particles through change their location and updating their velocity in the research space^20,38^. Mathematically, the particles ${X}_{i}=({x}_{i1},{x}_{i2},\dots .,{x}_{iD})$ and these situations with best fitting can be represented in the best current and global positions, these are ${P}_{i}=({p}_{i1},{p}_{i2},\dots,{p}_{iD})$ and ${P}_{g}=({p}_{g1},{p}_{g2},\dots,{p}_{gD})$. These can be attained through the best fitting function $Pbest$, and the best global function $Gbest$, respectively^38^. The velocity of particles is represented as ${V}_{i}=({v}_{i1},{v}_{i2},\dots,{v}_{iD})$. The following equations represent the velocity and position of particles updating in each iteration^38^:

7 $${V}_{id}(t+1)=\omega {V}_{id}\left(t\right)+{c}_{1}{r}_{1}\left({P}_{id}\left(t\right)-{X}_{id}\left(t\right)\right)+{c}_{2}{r}_{2}\left({P}_{gd}\left(t\right)-{X}_{id}\left(t\right)\right)$$

8 $${X}_{id}\left(t+1\right)={X}_{id}\left(t\right)+{V}_{id}\left(t+1\right), \,\,\,\, d=\mathrm{1,2},\dots .,D$$

where, ${r}_{1} \, {\text{and}} \, {r}_{2} {\text{are}} \, {\text{random}} \, {\text{values}} \, \epsilon (\mathrm{0,1})$, ${c}_{1} \, {\text{and}} \, {c}_{2}$ are acceleration coefficients, and $\omega$ is the inertia-weight factor.

### Grey wolf optimization (GWO)

GWO is also a population solution that simulates the social behaviour of a grey wolf pack^44^. In GWO, four categories are divided, are $alpha, beta, delta \, {\text{ and}} \, omega$^36^. The leaders of the whole pack represent as $alpha$ category (higher category). $Beta$ group helps the leader’s group in implementing commands on other lower categories. $Delta$ group uses to fulfil above commands and controls $omega$. The $omega$ group mainly follows all leaders’ commands by superior departments. The hunting plan contains three steps, that are identifying and chasing the prey, encircling and harassing prey until it stops resilience, and attacking on prey^36,44^.

The GW population is assumed in the optimization process as *n* with unknown *d*-dimensional search space^36^. GW positions can be expressed as ${X}_{wi}=[{x}_{i}^{1},{x}_{i}^{2},\dots ., {x}_{i}^{d}]$. The best fitting solution of $alpha, beta, \, {\text{and}} \, delta$ can be considered as ${X}_{\alpha }, {X}_{\beta }, \, {\text{and}} \, {X}_{\delta }$, respectively.

The hunting process, mathematically, can be modelled as follows:

9 $$\vec{D}=|\vec{C} \cdot {\vec{X}}_{p}\left(t\right)-{\vec{X}}_{w}\left(t\right)|$$

10 $${\vec{X}}_{w}\left(t+1\right)={\vec{X}}_{p}\left(t\right)+\vec{A} \cdot \vec{D}$$

where, ${\vec{X}}_{p} \, {\text{and}} \, {\vec{X}}_{w}$ are the position of prey and wolf, respectively; and $\vec{A} \, {\text{and}} \, \vec{C}$ are coefficients and can be calculated as:

11 $$\vec{A}=2\vec{a} \cdot {\vec{r}}_{1}-\vec{a}, \,\,\,\,\, \vec{C}=2{\vec{r}}_{2}$$

where, ${\vec{r}}_{1} \, {\text{and}} \, {\vec{r}}_{2}\in (\mathrm{0,1})$; $\vec{a}$ is a coefficient decreases linearly from 0 to 2 with increasing the iteration numbers.

The best positions for the best solution in this optimization method are determined based on the hunting process. The $alpha$ guidance performs in the hunting process. $Beta \, {\text{and}} \, delta$ might follow $alpha$ in trapping a prey to find the best solution for the prey. Then the $omega$ are pressed to follow and update positions according to the best positions of $alpha, beta, \, {\text{and}} \, delta$. Mathematically, the positions can be expressed as follows^36^:

12 $$\left.\begin{array}{c}{\vec{X}}_{1}={\vec{X}}_{\alpha }-{\vec{A}}_{1}.{\vec{D}}_{\alpha }\\ {\vec{X}}_{2}={\vec{X}}_{\beta }-{\vec{A}}_{2}.{\vec{D}}_{\beta }\\ \begin{array}{c}{\vec{X}}_{3}={\vec{X}}_{\delta }-{\vec{A}}_{3}.{\vec{D}}_{\delta }\\ \vec{X}(t+1)=\frac{{\vec{X}}_{1}+{\vec{X}}_{2}+{\vec{X}}_{3}}{3}\end{array}\end{array}\right\}$$

### Symbiotic organisms search (SOS)

SOS mimics three symbiotic interactions during searching (moving of ecosystems, population, organism, select solution) to find the best solution: mutualism, commensalism, and parasitism symbiosis’s^45^. The three symbiotic interactions can be summarized as follows^23,45^:

**(a)** The interactions of an organism with another organism are commonly beneficial in the symbiotic mutualism stage. This stage can be expressed as follows:

13 $$\left.\begin{array}{c}{x}_{i new}={x}_{i}+rand\left(\mathrm{0,1}\right).[{x}_{best}-\left(\frac{{x}_{i}+{x}_{ii}}{2}\right).(1+round\left(rand\left(\mathrm{0,1}\right)\right))]\\ {x}_{ii new}={x}_{ii}+rand\left(\mathrm{0,1}\right).[{x}_{best}-\left(\frac{{x}_{i}+{x}_{ii}}{2}\right).(1+round\left(rand\left(0,1\right)\right))]\\ \begin{array}{*{20}l}{x}_{i}=\left\{\begin{array}{*{20}l}{x}_{i} & f({x}_{i})\le f({x}_{i new})\\ {x}_{i \, new } & f\left({x}_{i}\right)>f({x}_{i \, new})\end{array}\right.\\ {x}_{ii}=\left\{\begin{array}{*{20}l}{x}_{ii} & f({x}_{ii})\le f({x}_{ii \, new})\\ {x}_{ii \, new } & f\left({x}_{ii}\right)>f({x}_{ii \, new})\end{array}\right.\end{array}\end{array}\right\}$$

where, ${x}_{i} and {x}_{ii}$ represent the *i*th and *ii*th organism vectors of the ecosystems in i and ii, $i\ne ii$. ${x}_{best}$ denotes the best organism in the current iteration, ${x}_{i new}$ and ${x}_{ii new}$ denote the respective organism for ${x}_{i} and {x}_{ii}$ after their interaction, and $f$ is the fitness function.

**(b)** In the commensalism stage, the interactions of an organism with another one benefit that organism and possess no effect on the other organism. This can be mathematically expressed as follows:

14 $$\left.\begin{array}{c}{x}_{i new}={x}_{i}+rand\left(-\mathrm{1,1}\right).({x}_{best}-{x}_{ii})\\ {x}_{i}=\left\{\begin{array}{*{20}l}{x}_{i} & f({x}_{i})\le f({x}_{i new})\\ {x}_{i \, new } & f\left({x}_{i}\right)>f({x}_{i \, new})\end{array}\right.\end{array}\right\}$$

**(c)** In the parasitism phase, the interactions of an organism with another one benefit that organism and harm the other organism, and this can be expressed as:

15 $$\left.\begin{array}{c}{x}_{parasite}=\left\{\begin{array}{*{20}l}{x}_{i} & if rand\left(\mathrm{0,1}\right)\le rand(\mathrm{0,1}) \\ LB+rand\left(\mathrm{0,1}\right).\left(UB-LB\right) & if rand\left(\mathrm{0,1}\right)>rand(\mathrm{0,1})\end{array}\right.\\ {x}_{ii}=\left\{\begin{array}{*{20}l}{x}_{ii} & f({x}_{ii})\le f({x}_{ii parasite})\\ {x}_{ii \, parasite } & f\left({x}_{ii}\right)>f({x}_{ii \, parasite})\end{array}\right.\end{array}\right\}$$

where, ${x}_{parasite}$ is the artificial parasite organism generated to compete with ${x}_{ii}$; LB and UB represent the lower and upper bound of the problem.

### Salp swarm algorithm (SSA)

SSA is a new population solution that simulates the behaviour of slaps in oceans throughout locomotion for finding the best solution to optimization problems^46,47^. The details of this method can be found in^48^. The summary of this method can be proposed as follows^37^: slaps divided into leaders and followers to mimic the best position solution. The followers follow the leader in the chain. The best solution can be found through the leader who is the front in the chain using the model up to a particular iteration. For *n* problem variables, the position of the slaps is saved in the *n*-dimension search space. This can be stored in a two-dimension matrix $k$, which presents the position of the leader and it can be expressed as follows:

16 $${k}_{j}^{1}=\left\{\begin{array}{*{20}l}{P}_{j}+{d}_{1}\left({u}_{j}-{l}_{j}\right){d}_{2}+{l}_{j}) & {d}_{3}\ge 0\\ {P}_{j}-{d}_{1}\left({u}_{j}-{l}_{j}\right){d}_{2}+{l}_{j}) & {d}_{3}\ge 0\end{array}\right.$$

where, *P* represents the food source, and ${P}_{j}$ is the position of food in the *j*th dimension. ${u}_{j}and {l}_{j}$ denote the upper and lower bounds of *j*th dimensions, respectively. ${d}_{2} and {d}_{3} \in (\mathrm{0,1})$, and ${d}_{1}$ represents a coefficient can be determined as:

17 $${d}_{1}=2{e}^{-{(4t/T)}^{2}}$$

where $t \, {\text{and}} \, T$ are the current and total number iterations, respectively. Here, the updating of the follower’s position after each iteration can be calculated based on Newton’s law of motion as:

18 $${k}_{j}^{i}=0.5a{t}^{2}+{v}_{0}t$$

where ${k}_{j}^{i}$ represents the *i*th follower particle in the *j*th dimension; where, *i* = 1 for the leader position (Eq. 16), and $i\ge 2$ is for the followers. $t$ is time, ${v}_{0}$ denotes the beginning velocity, and $a=\frac{{v}_{final}}{t};and {v}_{final}=\frac{k-{k}_{0}}{t}$. By considering ${v}_{0}=0$ and substituting these values in Eq. 18, the updating followers slaps can be presented as:

19 $${k}_{j}^{i}=0.5({k}_{j}^{i}+{k}_{j}^{i-1})$$

Then, the Eqs. 16 and 19 can be utilized to update the slaps at each iteration in SSA for optimizing the problem variables.

### Slime mould algorithm (SMA)

The SMA is one of the new swarm meta-heuristic algorithms; it mathematically mimics the propagation wave of slime mould when simulating the best path for connecting foods^39,49^. The details of this method are in 30, and it is proposed in two stages: approaching and warp foods. The details of both stages can be summarized as follows^49^:

**(a)** Approaching food stage: the slime approaching food in this phase based on its odour in the air, and this can be mathematically expressed as follows:

20 $$\vec{X}\left(t+1\right)=\left\{\begin{array}{*{20}l}{\vec{X}}_{b}\left(t\right)+\vec{vb}.\left(\vec{W}.{\vec{X}}_{A}\left(t\right)-{\vec{X}}_{B}\left(t\right)\right), & r<P\\ \vec{vc}.\vec{X}\left(t\right), & r\ge P\end{array}\right.$$

where, $X$ represents the position of the slim mould, ${X}_{A} and {X}_{B}$ are randomly selected from the mould, ${X}_{b}$ is the current position related to high odour concentration. *t* is the current iteration, $vc$ is a parameter gradually decreasing from 1 to zero in a linear form, $vc$ is a parameter ranges from $a$ to − $a$, where $a=\mathrm{arctanh}(-\left(\frac{t}{\mathrm{max}\left(t\right)}\right)+1)$. W is the weight of the slim mould and it can be generated and updated based on the fitting accuracy, see^39^. $r\in (\mathrm{0,1})$, and $P=\mathrm{tanh}\left[S\left(i\right)-DF\right], i=\mathrm{1,2},3,\dots,n$, here, $S\left(i\right)$ is the fitness values of *X*, and *DF* is the best fitness over the whole iterations.

**(b)** Warp food stage: in this stage, the slime behaviour of venous structures can be expressed as:

21 $${\vec{X}}^{*}=\left\{\begin{array}{*{20}l} rand.\left(UB-LB\right)+LB, & rand<z\\ {\vec{X}}_{b}(t)+\vec{vb}.\left(\vec{W}.{\vec{X}}_{A}(t)-{\vec{X}}_{B}(t)\right), & r<P\\ \vec{vc}.\vec{X}\left(t\right), & r\ge P\end{array}\right.$$

where, LB and UB represent the lower and upper bound of the search range. $rand$ is the random value in between 0 and 1.

### Harris hawks optimization (HHO)

HHO has been developed by Heidari et al.^50^ as a new optimization technique. It applies a resemblance of Harris hawks cooperative behaviour in optimization solutions^38,50^. The details of this technique can be found in^50^. In general, HHO depends on three phases that are exploration, transferring, and exploiting^38^. In exploration stage, the position of hawks is determined using the following equation^38^:

22 $$\Upsilon \left( {iter + 1} \right) = \left\{ {\begin{array}{*{20}l} {\Upsilon _{{rand}} \left( {iter} \right) - r_{1} |\Upsilon _{{rand}} \left( {iter} \right) - 2r_{2} \Upsilon \left( {iter} \right)} \hfill & {if\;n \ge 0.5} \hfill \\ {\left( {\Upsilon _{{prey}} \left( {iter} \right) - \Upsilon _{m} (iter)} \right) - r_{3} \left( {LB + r_{4} \left( {UB - LB} \right)} \right)} \hfill & {if\;n < 0.5} \hfill \\ \end{array} } \right.$$

where, ${\Upsilon }_{rand}$ and ${\Upsilon }_{prey}$ represent randomly selection hawks and prey’s position, respectively. ${r}_{i}$ denotes a random value in between 0 to 1. ${\Upsilon }_{m}$ is the average position.

In transferring phase, the prey energy can be modelled as $=2{E}_{0}(1-(iter/T))$, where ${E}_{0} \, {\text{and}} \, T \epsilon (-\mathrm{1,1})$, and by determining E, the hawk decides whether to search for exploit the neighbourhood of the solutions. In short, beginning the exploration phase when $|E|\ge 1$, and exploiting the neighbourhood when $\left|E\right|<1$. Based on the exploration phase, hawks select soft or hard besiege in applying.

## Design algorithms and evaluation

### Integration models designs

In the present study, six meta-heuristic OAs, namely PSO, GWO, SOS, SSA, HHO, and SMA are used to optimize the hyper-parameter of LSSVM. These hyper-parameters are $\gamma$ and $\sigma$. Note that the model establishment of LSSVM requires an appropriate setting of its hyper-parameters, including their regularization for constructing an optimum model. The hyper-parameter $\gamma$ (regularization parameter) and $\sigma$ (kernel parameter) strongly affect the performance of the LSSVM model, and hence they should be tuned properly for constructing the optimum model in predicting the desired output. In addition to the hyper-parameter, the selection of kernel function also plays an important role. Therefore, proper selection of hyper-parameters of LSSVM at one go is not a trivial task because they should be searched in continuous domains, and hence, there will be an infinite number of parameters sets. Thus, the problem of parameter tuning of LSSVM can be formulated as an optimization problem, and a meta-heuristic OA can solve this.

**Figure 1:**
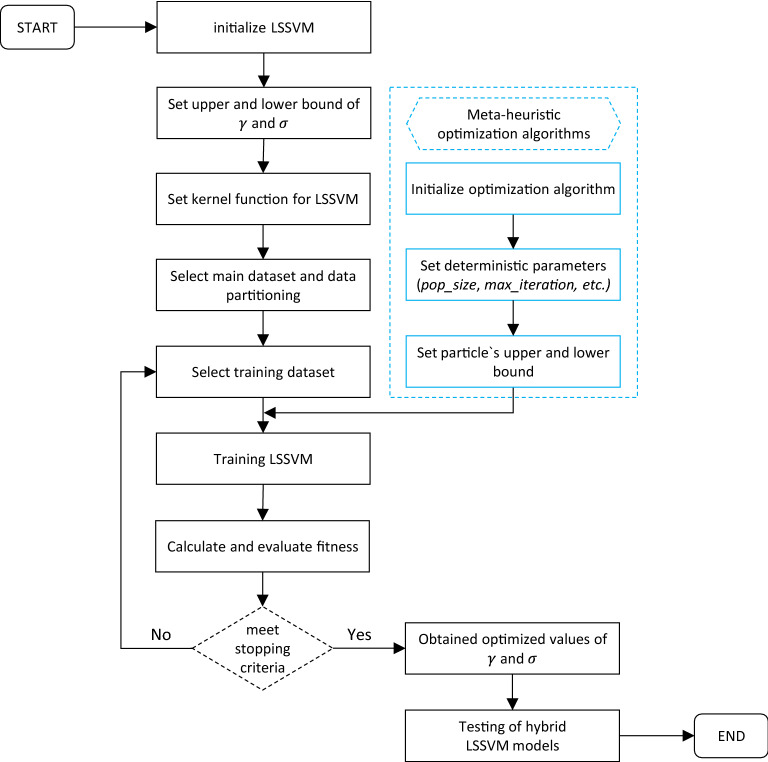
A flowchart showing the steps of hybrid LSSVM models.

Considering the above points as a reference, PSO, GWO, SOS, SSA, HHO, and SMA were used to optimize $\gamma$ and $\sigma$ of LSSVM and six hybrid LSSVM models LSSVM-PSO, LSSVM-GWO, LSSVM-SOS, LSSVM-SSA, LSSVM-HHO, and LSSVM-SMA, were constructed. The steps of optimizing LSSVM parameters using OAs can be described as follows: (a) initialize LSSVM, (b) set upper and lower bounds of $\gamma$ and $\sigma$, (c) set kernel function, (d) data partitioning, (e) selection of training dataset, (f) initialize OAs, (g) set deterministic parameters of OAs such as, swarm size (n_s_), number of iterations (i_max_), upper and lower bounds (UB and LB), etc., (h) training of LSSVM algorithm, (i) calculate the fitness function, (j) check and evaluate fitness, (k) obtained optimized values of $\gamma$ and $\sigma$, and (l) testing of hybrid LSSVMs based on obtained values of $\gamma$ and $\sigma$. Figure 1 presents the steps of developing hybrid LSSVM models in the form of a flow chart. Note that, apart from hyper-parameters of LSSVM, the deterministic parameters of OAs also play an important role in hybrid modeling; therefore, they should be tuned appropriately during the course of the optimization process.

### Statistical and uncertainty models evaluations

To evaluate the accuracy of the proposed models, different statistical parameters were applied and evaluated. Correlation statistical parameters, viz., determination coefficient (R^2^), Nash–Sutcliffe efficiency (NS), and variance account factor (VAF) were calculated to assess the linearity correlation between observed and predicted Mr. Here, when three parameters’ values are equal, it means the accuracy of the model is high^51^. Error statistical parameters, viz., root mean square error (RMSE), mean absolute error (MAE), and root mean square error to observation’s standard deviation Ratio (RSR), were determined to assess the models’ errors. The percentage of error (PE) was calculated to evaluate the model accuracy in predicting Mr. These are the widely used performance indices^10,51–55^, the mathematical expressions of which can be given by:

23 $${R}^{2}=\frac{{\sum_{i=1}^{n}(Mrm-\overline{Mr }m)}^{2}-\sum_{i=1}^{n}{(Mrm-Mrp)}^{2}}{{\sum_{i=1}^{n}(Mrm-\overline{Mr }m)}^{2}}$$

24 $$NS=1-\frac{\sum_{i=1}^{n}{(Mrm-Mrp)}^{2}}{{\sum_{i=1}^{n}(Mrm-\overline{Mr }m)}^{2}}$$

25 $$VAF=1-\frac{var(Mrm-Mrp)}{var(Mrm)}$$

26 $$RMSE=\sqrt{\frac{\sum_{i=1}^{n}{(Mrm-Mrp)}^{2}}{n}}$$

27 $$MAE=\frac{1}{n}\sum_{i=1}^{n}|Mrm-Mrp|$$

28 $$RSR=\frac{RMSE}{\sqrt{\frac{1}{n}{\sum_{i=1}^{n}(Mrm-\overline{Mr }m)}^{2}}}$$

29 $$PE=\frac{RMSE}{Mrmax-Mrmin}\times 100$$

where $Mrm \, {\text{and}} \, \overline{Mr }m$ represent the measured and mean of measured Mr, respectively; $Mrp$ denotes the predicted Mr. $Mrmax \, {\text{and}} \, Mrmin$ are the maximum and minimum measured Mr, respectively, and *n* is the dataset number.

In addition, Visual evaluation was presented and discussed. In the current study, three visual statistical methods were applied: regression error characteristics (REC) curve, rank analysis, and violin plot. REC curve measures the model accuracy based on the amount of error in the form of squared residuals. The cumulative distribution function of the error between the actual and predicted values is used to determine the model accuracy. The area over the curve (AOC) represents the performance of model accuracy, the model that has a small AOC value is the best model. The rank analysis is another simple visual evaluation technique, it depends on the statistical indices, Eqs. 23–29. In this analysis, the models score a rank from 1 to 6; rank 1 indicates low performance and six refers to the model with good modeling performance. The total rank for the training and testing phases implies which model is the best for modelling the Mr of subgrade soils. Violin plot is another visual analysis technique, it is similar to the box-plot with showing the distribution of probability density of the measured values. This study presented the violin plot for models’ errors to compare the best proposed models in the error evaluation. More details for these methods can be found in^55,56^.

For more investigation, the reliability of each proposed models in measuring Mr was assessed through the uncertainty analysis (UA) index. The UA of models can be utilized to test the proposed models under different experimental conditions. Here, for the model error (E_i_), the mean of error (MOE) and standard deviation (SD) can be calculated as follows:

30 $${E}_{i}=\left|{O}_{i}-{P}_{i}\right|; MOE=\frac{1}{n}\sum_{i=1}^{n}{E}_{i}; SD=\sqrt{\frac{\sum_{i=1}^{n}{({E}_{i}-\overline{E })}^{2}}{n-1}}$$

Moreover, the standard and margin errors, i.e., SE and ME, respectively, are used to calculate the width of the confidence bounds (WCB)^57^. The WCB indicates the upper bound (UB) and lower bound (LB) uncertainty of the proposed models, and it can be determined as:

31 $$WCB=\frac{t.SD}{\sqrt{n}}$$

where *t* denotes the left-tailed inverse of the error distribution. The 95% confidence interval of prediction error can be determined using the values of WCB and MOE. The UB and LB indicate the error range in which approximately 95% of data are located. The lower UA statistical indices, the greater model certainty, indicating a small error in predicting Mr values.

### Sensitivity analysis

To determine the impact of input variables in modelling Mr, the sensitivity of these inputs was conducted. This can provide a guide for using/un-using these variables in the proposed models based on the significance of each variable on the predicted value of Mr. This may help decrease the complexity of proposed models and decrease the measurement cost in future applications. In this study, the cosine amplitude method (CAM) was implemented to assess the impact of input variables^55^. Based on CAM, the strength between Mr and input variables can be determined as follows:

32 $${R}_{ij}=\frac{\sum_{k=1}^{m}{x}_{ik}{x}_{jk}}{\sqrt{\sum_{k=1}^{m}{{x}_{ik}}^{2} \sum_{k=1}^{m}{{x}_{jk}}^{2}}}\times 100$$

where data pairs, ${x}_{i} \, {\text{and}} \, {x}_{j}$, of datasets are constructed to measure the strength of the relation. The closer ${R}_{ij}$ to 100 means more impact corresponding variable has on the Mr value.

## Soil sites and datasets

**Figure 2:**
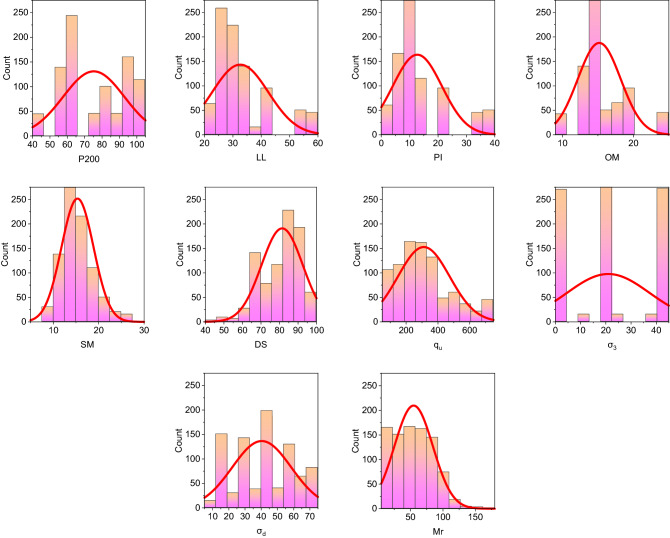
Histogram and normal distribution of datasets of subgrade soil variables.

The used dataset of soils in this study was collected from literature presented in the Ohio Department of Transportation^3^. The data were collected from different road construction sites. A total of 891 datasets composed of three types (418 datasets for A-4, 283 datasets for A-6, and 190 datasets for A-7) of subgrade material in Ohio, which are cohesive soils, were used in this study to predict the Mr using the proposed models. The dry side of the optimum, optimum, wet side of optimum, and saturated moister content of soil water conditions were considered in Mr tests, which were performed according to AASHTO standardization^4^. Hanittinan^3^ found nine input variables that affect the modeling of Mr, which are percent of soil particles passing through #200 sieve (P200) (fines content), liquid limit (LL), plasticity index (PI), optimum moisture content (OM), soil moisture content (SM), degree of soil saturation (DS), unconfined compressive strength (${q}_{u}$), confining stress (${\sigma }_{3}$) and deviator stress (${\sigma }_{d}$). Table 1 presents the statistical analysis (maximum (Mx), minimum (Mn), average (M), and standard deviation (SD)) of these variables for the whole datasets, and Fig. 2 demonstrates the histogram and normal distribution of them.

**Table 1:** Statistical characteristics of main engineering properties of soil datasets.

Indices	P#200	LL	PI	OM (%)	SM (%)	DS (%)	${q}_{u}$ (kPa)	${\sigma }_{3}$ (kPa)	${\sigma }_{d}$ (kPa)	Mr (MPa)
Mx	100.0	59.0	36.0	24.2	27.2	100.0	715.7	41.4	71.7	179.4
Mn	42.0	21.0	2.0	9.4	7.5	42.9	54.3	0.0	10.0	6.4
M	75.2	32.6	12.7	15.2	15.3	81.4	311.1	21.0	40.3	54.8
SD	17.6	9.9	8.7	3.0	3.5	11.2	162.8	16.4	18.2	29.7

From Table 1, it can be seen that the variation between datasets for one variable is high. In addition, from Table 1 and Fig. 2, it can be shown that the distribution of the variables is almost not normal. It can be noted from Table 1 and Fig. 2 a significant variation in the range of dataset for each variable can be detected. This means that a non-linear relationship between inputs and output variables can be detected, and this gives an advantage for using hybrid soft computing techniques in this study compared to traditional techniques.

## Results and discussion

### Parametric configuration of the developed hybrid models

As stated earlier, the selection of hyper-parameters of LSSVM and deterministic parameters of OAs play an important role for constructing the optimum model, therefore, the values of two hyper-parameters ($\gamma$ and $\sigma$) were set within a pre-defined wide range of upper and lower bounds. In this study, these parameters' upper and lower bounds were set to (100 and 0.10) and (50 and 0.10), respectively. In each iteration of hybrid LSSVM models, the two hyper-parameters of LSSVM were randomly generated within the range of upper and lower bounds utilizing the following equation:

33 $$\gamma \, {\text{and}} \, \sigma =rand\times \left(UB-LB\right)+LB$$

where UB and LB are the upper and lower bounds of hyper-parameters, $rand$ is a uniformly distributed random number generated within the range of 0 to 1. On the other hand, three different sets of deterministic parameters of OAs were investigated to ensure effective selection of hybrid LSSVM models.

To construct the optimum hybrid models of LSSVM (i.e., LSSVM-PSO, LSSVM-GWO, LSSVM-SOS, LSSVM-SSA, LSSVM-HHO, and LSSVM-SMA), the value of n_s_ was set to 25, 50, and 100, whereas the i_max_ = 100 was set in each case. The values of exploration and exploitation constants and other deterministic parameters were kept at their original values, as proposed in the original studies of PSO, GWO, SOS, SSA, HHO, and SMA. For instance, the exploration and exploitation constants of PSO were set to 1 and 2, respectively. The value of parameter z in LSSVM-SMA was set to 0.03. It is worth noting that, prior to constructing the models, the main dataset was partitioned into training and testing subsets; among them, the training subset was used to construct the hybrid models, while the testing subset was used to assess the predictive capability of the constructed LSSVM models. The detailed hybrid models constructed with three sets (Set 1, Set 2, and Set 3) are presented in Table 2 for training and training subsets. Herein, the RMSE values of developed models are given in terms of normalized predicted outputs.

**Table 2:** RMSE value for different sets of constructed models.

Models	Set 1 (n_s_ = 25)		Set 2 (n_s_ = 50)		Set 3 (n_s_ = 100)	
	Training	Testing	Training	Testing	Training	Testing
LSSVM-PSO	0.0155	0.0687	0.0146	**0.0685**	0.0180	0.0688
LSSVM-GWO	0.0090	0.0433	0.0068	**0.0429**	0.0128	0.0431
LSSVM-SOS	0.0125	0.0417	0.0112	**0.0414**	0.0154	0.0419
LSSVM-SSA	0.0089	0.0432	0.0068	**0.0428**	0.0129	0.0437
LSSVM-HHO	0.0207	0.0516	0.0199	**0.0511**	0.0226	0.0513
LSSVM-SMA	0.1070	0.1222	0.1070	**0.1215**	0.1071	0.1221

Significant values are in bold.

**Figure 3:**
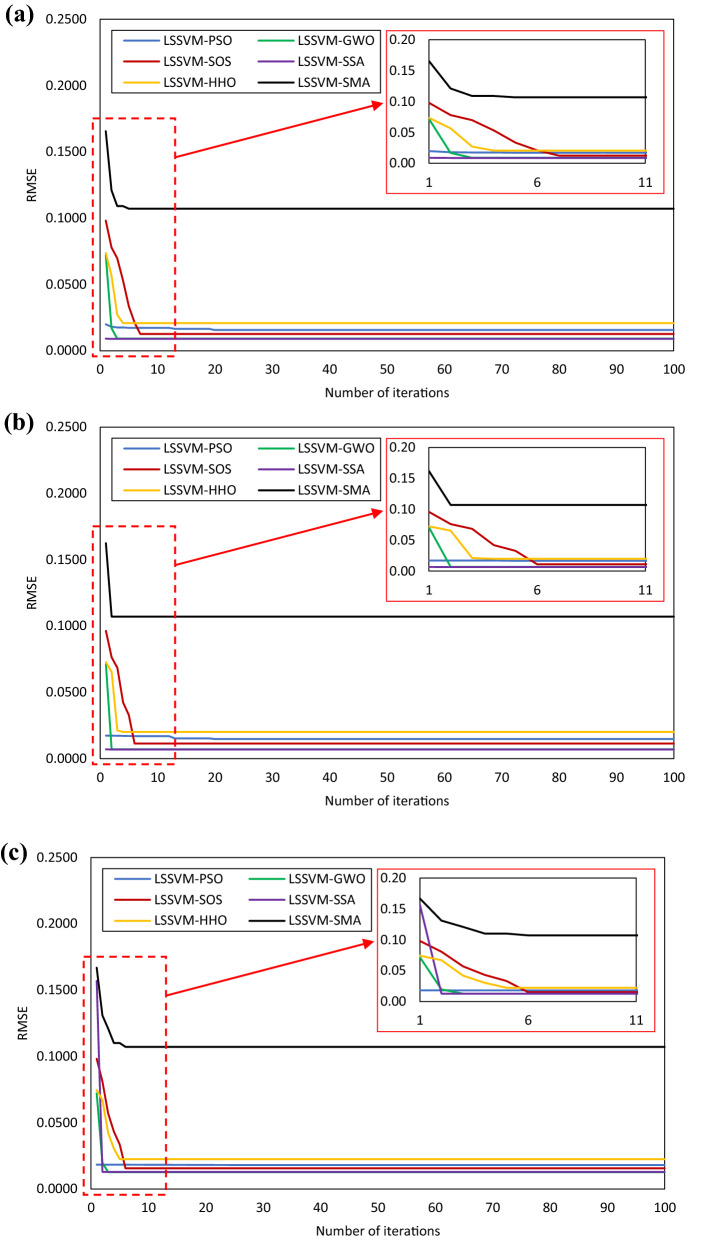
Convergence behaviour of hybrid LSSVMs for (**a**) Set 1, (**b**) Set 2, and (**c**) Set 3.

Right after the model development, they were assessed based on the performance of the testing dataset. It is pertinent to mention that, a model that attained higher prediction accuracy in the testing phase should be accepted with more conviction. As can be seen, all the developed models attained the most accurate prediction when the value of n_s_ was set to 50. Form the results presented in Table 2, it can also be observed that the developed LSSVM-SOS attained the most accurate prediction in the testing phase in all cases, indicating high generalization ability. However, the details of n_s_, i_max_, UB and LB of $\gamma$ and $\sigma$, cost function, and the optimum values of $\gamma$ and $\sigma$ are presented in Table 3. Herein, the values of these parameters are presented for Set 2 combination of hybrid LSSVM model construction. In addition, the convergence behaviour of three different combinations is presented in Fig. 3. As can be seen, the developed hybrid models converge in less than 20 iterations indicating lower computation cost in all cases. Note that, all the hybrid models were constructed in MATLAB 2015a environment. In the following sub-section, the outcomes of the developed models in predicting Mr of subgrade soils are presented, analyses, and compared.

**Table 3:** Configuration of OAs for Set 2 hybrid LSSVMs.

Parameters	LSSVM-PSO	LSSVM-GWO	LSSVM-SOS	LSSVM-SSA	LSSVM-HHO	LSSVM-SMA
n_s_	50	50	50	50	50	50
i_max_	100	100	100	100	100	100
UB of $\gamma$,$\sigma$	100, 100	100, 100	100, 100	100, 100	100, 100	100, 100
LB of $\gamma$,$\sigma$	0.10, 0.10	0.10, 0.10	0.10, 0.10	0.10, 0.10	0.10, 0.10	0.10, 0.10
Cost function	RMSE	RMSE	RMSE	RMSE	RMSE	RMSE
$\gamma$	0.05	100	100	99.99	9.78	0.10
$\sigma$	9.49	0.01	1.12	0.10	0.01	0.10

### Models’ performances

Table 4 presents the statistical evaluation of the proposed models. The correlation between the actual and predicted Mr values is high for all models except LSSVM-SMA model in the training and testing stages. The three statistical correlation parameters (R^2^, NS and VAF) are seen equal for models LSSVM-GWO, LSSVM-SOS, and LSSVM-SSA in the training stage. Furthermore, the model error parameters (RMSE, MAE and RSR) are shown small for the same models in the training (tr) and testing (ts) stages. This means that the accuracy of LSSVM-GWO, LSSVM-SOS, and LSSVM-SSA in modelling Mr is high and can be used as the best models. The percentage error of LSSVM-SOS model is shown low in the training (5.613%) and testing (13.346%) phases. In addition, the statistical parameters of correlation and model errors are shown the best for LSSVM-SOS model in the testing stage, R^2^ = 0.942, RMSE = 6.724 MPa. These results imply that the accuracy of LSSVM-SOS is high and can be used to estimate Mr of subgrade soils.

**Table 4:** Statistical analyses of the proposed models.

Model	R^2^	NS	VAF	RMSE	MAE	RSR	PE
**Training phase**							
LSSVM-PSO	0.994	0.993	0.993	2.150	1.883	0.072	7.343
LSSVM-GWO	**0.998**	**0.998**	**0.998**	**1.157**	**0.705**	**0.039**	6.272
LSSVM-SOS	0.996	0.996	0.996	1.888	1.293	0.064	**5.613**
LSSVM-SSA	**0.998**	**0.998**	**0.998**	**1.162**	**0.709**	**0.039**	6.289
LSSVM-HHO	0.988	0.987	0.987	3.162	2.499	0.106	8.955
LSSVM-SMA	0.752	0.611	0.611	7.283	15.586	0.245	50.101
**Testing phase**							
LSSVM-PSO	0.863	0.839	0.844	8.713	7.702	0.294	53.846
LSSVM-GWO	0.940	0.937	0.938	6.789	4.790	0.229	27.847
LSSVM-SOS	**0.942**	**0.941**	**0.942**	**6.724**	**4.399**	**0.227**	**13.346**
LSSVM-SSA	0.940	0.937	0.939	6.781	4.785	0.229	27.713
LSSVM-HHO	0.920	0.911	0.913	7.392	5.992	0.250	33.698
LSSVM-SMA	0.632	0.494	0.496	7.666	16.655	0.259	60.898

Significant values are in bold.

**Figure 4:**
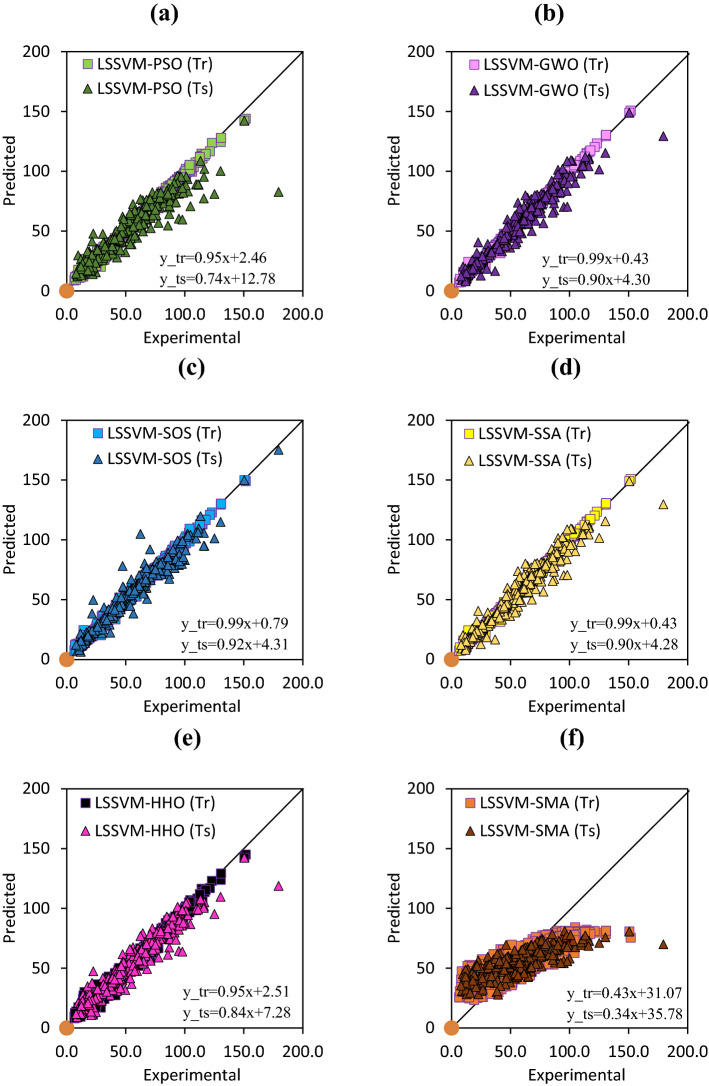
Proposed models, (**a**) PSO, (**b**) GWO, (**c**) SOS, (**d**) SSA, (**e**) HHO, (**f**) SMA, performances in the training (Tr) and testing (Ts) stages.

For further investigations, a scatter plot of model’s performances in the training and testing phases is presented in Fig. 4. The linear fitting equation is presented to show the overfitting performance of the proposed models. The comparison between the presented models shows that the performance of LSSVM-SMA is low, and it is the worst in modelling Mr values. Other model’s overfittings are acceptable. The slopes of the linear fitting of LSSVM-PSO in the training and testing stages are 0.95 and 0.74, respectively. This means that the overfitting of this model is high. The slopes for LSSVM-HHO are 0.99 and 0.84 in the training and testing stages, respectively. The slopes for LSSVM-GWO are 0.99 and 0.90 in the training and testing stages, respectively, and for LSSVM-SOS are 0.99 (training) and 0.92 (testing), and for LSSVM-SSA are 0.99 and 0.90 for the training and testing stages, respectively. This means that the LSSV-SOS model's overfitting is lower than other proposed models. So, the accuracy of LSSVM-SOS is high in predicting the Mr values.

### Visual and uncertainty models evaluations

**Figure 5:**
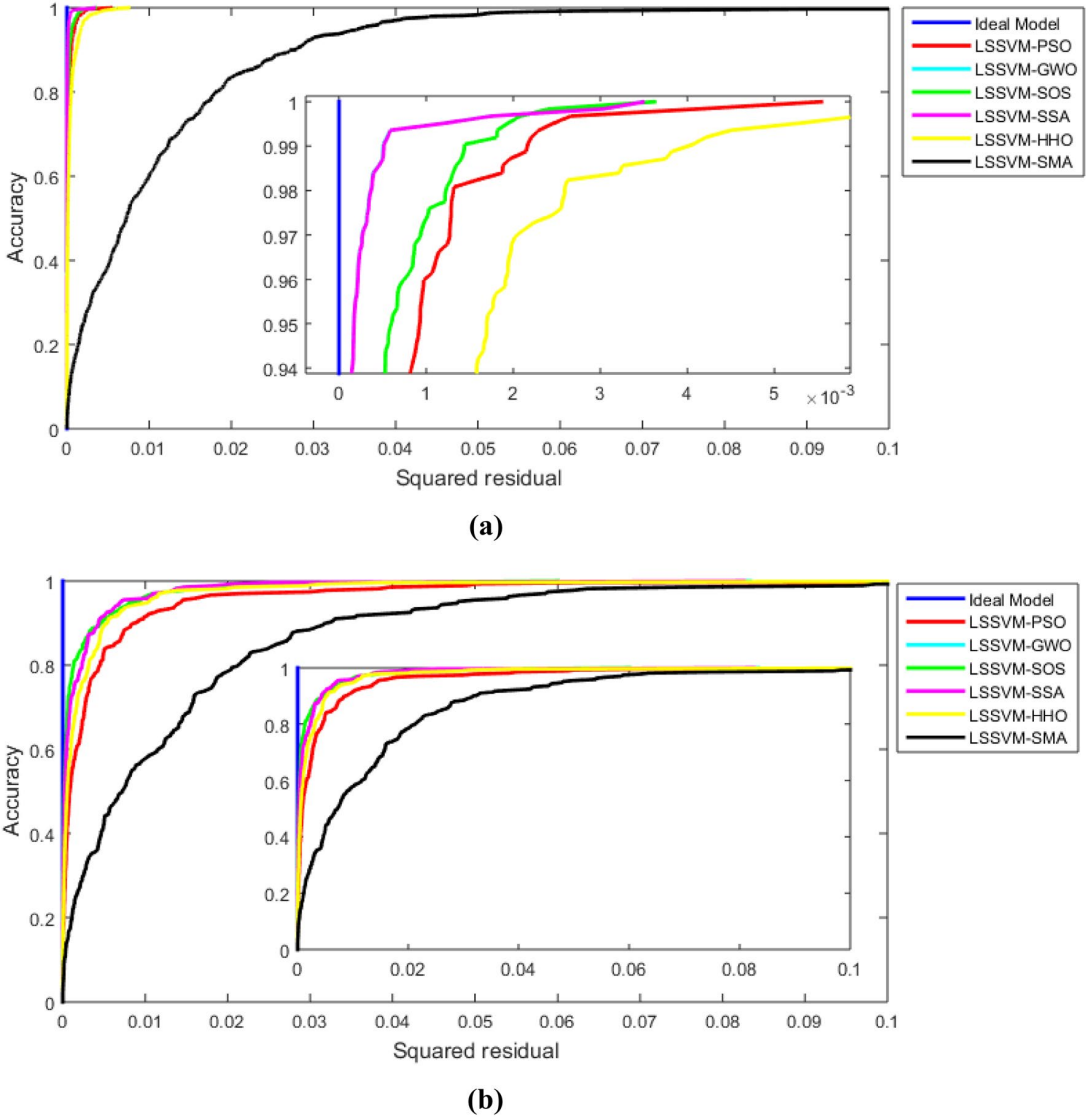
REC curve for: (**a**) training and (**b**) testing datasets.

Figure 5 illustrates the REC curves for the training and testing stages, and Table 5 presents the AOC of the proposed models. From Fig. 5 and Table 5, it is obviously shown that the accuracy of LSSVM-GWO and LSSVM-SSA is high in the training stage, while the accuracy of LSSVM-SOS in the testing stage is high to predict the Mr value. The worst model in the training and testing stages is obviously the LSSVM-SMA model. The AOC values of LSSV-SMA in the training (0.0113) and testing (0.014) are shown high. The AOC value for the LSSVM-SOS, LSSVM-GWO, and LSSVM-SSA in the testing stage is 0.0016, 0.0017, and 0.0017, respectively. This means that these models' performances are high compared to other models, and the performance of LSSVM-SOS is the best in this study.

**Table 5:** AOC values for the REC of the proposed models.

Actual/models	Training	Testing
Actual	0.000000	0.000000
LSSVM-PSO	0.000210	0.004100
LSSVM-GWO	**0.000044**	0.001700
LSSVM-SOS	0.000122	**0.001600**
LSSVM-SSA	**0.000044**	0.001700
LSSVM-HHO	0.000390	0.002400
LSSVM-SMA	0.011300	0.014000

Significant values are in bold.

The rank analysis is proposed in Table 6. It can be seen from the table that the rank analyses of LSSVM-PSO (33), LSSVM-HHO (35), and LSSVM-SMA (16) are low compared to other proposed models. The rank of LSSVM-GWO is 41 in the training stage, while it is 28 in the testing stage. At the same time, the rank of LSSVM-SSA in the training and testing phases are 34 and 36, respectively. The LSSVM-SOS ranks in the training and testing stages are 30 and 42, respectively. The rank index of LSSVM-GWO, LSSVM-SOS, and LSSVM-SSA are 69, 72, and 70, respectively. This indicates that LSSVM-SOS outperformed other models in predicting Mr of subgrade soils, and it can be used as a soft computing technique for estimating the Mr values.

**Table 6:**
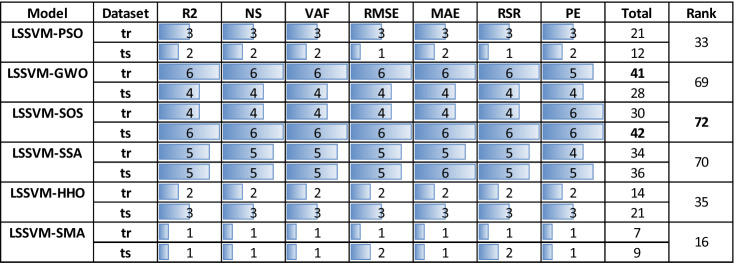
Model’s rank evaluation.

Significant values are in bold.

From REC and rank evaluations, it can be concluded that the LSSVM-GWO, LSSVM-SOS, and LSSVM-SSA are the best models and can be used in predicting the Mr of subgrade soils. The UA of the developed hybrid LSSVMs was performed and the results are presented in Table 7. To ensure the robustness of the proposed hybrid models, the UA was performed for the testing dataset only. From the information presented in Table 7, it can be seen that the LSSVM-SOS has lower MOE (0.0254), LB (0.215), and UB (0.0294) compared to the proposed models. This means that the accuracy of LSSVM-SOS model in predicting Mr is high at a confidence level of 95%. The SE, ME, and WCB of LSSVM-SOS, LSSVM-GWO and LSSVM-SSA models are the same, this indicates that the three models can be used to estimate the Mr with low uncertainty and high confidence level. A comparison of the whole UA is presented in the rank index; as presented in the table, it can be seen that the LSSVM-SOS has the lowers model errors, with rank 1, among the three models, followed by the proposed models. Therefore, LSSVM-SOS can be used to estimate accurate Mr values of subgrade soils.

**Table 7:** Results of UA.

Model	N	MOE	SD	SE	ME	LB	UB	WCB	Rank
LSSVM-PSO	267	0.0445	0.0520	0.0032	0.0063	0.0382	0.0508	0.0125	5
LSSVM-GWO	267	0.0277	0.0328	0.0020	0.0039	0.0237	0.0316	0.0079	3
LSSVM-SOS	267	0.0254	0.0327	0.0020	0.0039	0.0215	0.0294	0.0079	1
LSSVM-SSA	267	0.0277	0.0327	0.0020	0.0039	0.0237	0.0316	0.0079	2
LSSVM-HHO	267	0.0346	0.0376	0.0023	0.0045	0.0301	0.0392	0.0091	4
LSSVM-SMA	267	0.0962	0.0742	0.0045	0.0089	0.0873	0.1052	0.0179	6

**Figure 6:**
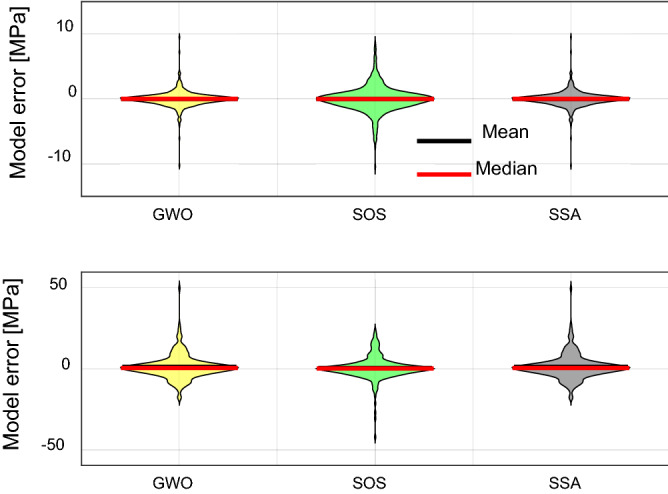
Violin plot for the proposed models in the training (upper) and testing (lower) stages.

Finally, violin plot is presented in Fig. 6 to present the model errors of LSSVM-GWO, LSSVM-SOS, and LSSVM-SSA in the training and testing stages. From Fig. 6, it can be seen that even the LSSVM-SOS model has a maximum error in the training stage, the model error distribution is shown normally. The mean and median of model’s errors are the same for the three models. In the testing stage, the maximum errors of models are observed in LSSVM-GWO and LSSVM-SSA. The shape of the violin plot is the same for both models. This means that the performance of both models is the same in predicting Mr. The maximum error of LSSVM-SOS is smaller than LSSVM-GWO and LSSVM-SSA, and the variation between the median and mean of the model error is shown smaller than other models. The model’s errors distributions are approximately the same for the three models. These results indicate that LSSVM-SOS is the best to use in predicting Mr of subgrade soils.

The developed LSSVM-SOS was compared to some of the previously proposed models designed to detect the Mr of subgrade soils and use the same input variables. Table 8 presents the overall performances of these models in terms R^2^. From Table 8, it can be seen that the new LSSVM-SOS outperforms other historical designed models, and it is a superior alternative to the traditional models in predicting the Mr of subgrade soils. The previous (ANN-GA) and current hybrid models have the same performance, in terms R^2^, accuracy in modelling the Mr with low complexity in the modelling calculations. However, the LSSVM-SOS model outperforms ANN-GA in terms RMSE. The overall RMSE of LSSVM-SOS and ANN-GA is 4.31 and 5.35, respectively. This means that LSSVM-SOS is more robustness and accuracy in estimating Mr of subgrade soils.

**Table 8:** Comparison LSSVM-SOS with prior models.

Model	Ref.	Inputs	R^2^
Soil-type model	^58^	P200, LL, PI, OM, SM, DS, ${q}_{u}$, ${\sigma }_{3}$,${\sigma }_{d}$	0.56
GA	^59^		0.87
LGP	^60^		0.83
ANN-GA	^59^		0.97
LSSVM-SOS	Current work		0.97

*GA* Genetic algorithm, *LGP* Linear genetic programming.

Herein, the main advantages of the proposed LSSVM-SOS model include (a) faster convergence (in less than 20 iterations), (b) lower computational cost, and (c) higher generalization ability. The selection of optimum values of $\gamma$ and $\sigma$ can also be solved through OAs. However, the proposed hybrid model has been investigated for a particular case of Mr prediction; therefore, further research should be carried out to ensure its robustness at all levels. The future direction of this study may include (a) a comprehensive assessment of the accuracy of LSSVM-SOS and other hybrid LSSVMs using other datasets from different fields; (b) a comparative assessment of LSSVM-SOS and other regression-based hybrid models such as relevance vector machine, Gaussian process regression, etc.; and (c) a comparative assessment of hybrid LSSVMs constructed with another group of OAs, such as evolutionary, physics-based OAs, etc.

### Variables impacts on Mr modelling

**Figure 7:**
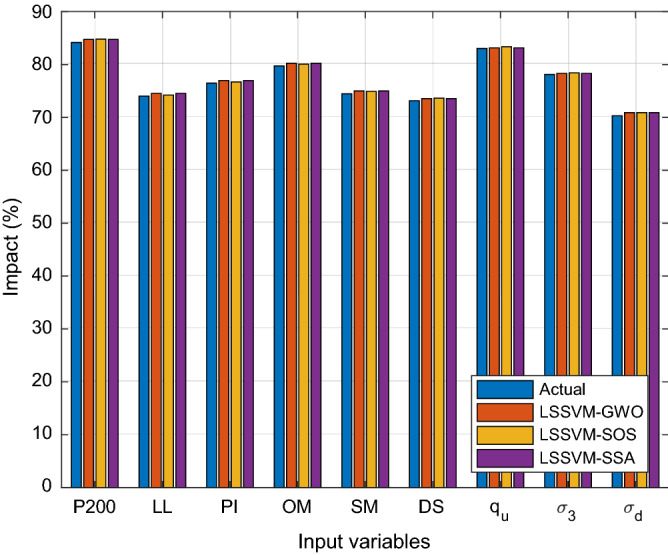
Impact of input variables on modelling Mr of subgrade soils.

For better assessment the performance of the developed hybrid models, sensitivity analysis was performed. As stated above, the cosine amplitude method (CAM)^62^ was used to perform the sensitivity analysis. Table 9 presents the outcomes of the sensitivity analysis for the different proposed models. In addition, the relative impact of input variables on Mr is presented in Fig. 7 for LSSVM-SOS, -GWO, and -SSA models. It is obviously observed that the impact of all variables in modelling and determining Mr is above 70% (refer to Fig. 7) which means that all variables possess a high impact in modelling Mr values. All variables have approximately the same the impact on the proposed models. Thus, the contribution of all variables cannot be neglected in modelling Mr of subgrade soils. However, the variables fine content (P#200), optimum moisture content, and unconfined compressive strength are shown more significant impact on the Mr, with an impact greater than 80%. These results are in agreement with state-of-the-art studies^58–61^.

**Table 9:** Results of sensitivity analysis for the developed hybrid LSSVMs.

Parameters	Actual	LSSVM-PSO	LSSVM-GWO	LSSVM-SOS	LSSVM-SSA	LSSVM-HHO	LSSVM-SMA
P#200	0.843	0.859	0.847	0.848	0.847	0.856	0.915
LL	0.740	0.757	0.746	0.742	0.746	0.753	0.802
PI	0.765	0.781	0.770	0.767	0.770	0.778	0.819
OM	0.797	0.815	0.802	0.801	0.802	0.810	0.886
SM	0.745	0.766	0.750	0.749	0.750	0.759	0.871
DS	0.732	0.752	0.736	0.737	0.736	0.744	0.872
q_u_	0.830	0.835	0.832	0.834	0.832	0.838	0.875
σ_3_	0.781	0.789	0.783	0.784	0.783	0.789	0.829
σ_d_	0.703	0.721	0.709	0.709	0.709	0.717	0.808

### Analysis of robustness of LSSVM-SOS

It is important to note that overfitting is a prevalent problem in data-driven modelling. It means that a data-driven or machine learning model can successfully estimate the desired output during both the training and testing phases, but it can also predict exceedingly odd results for datasets obtained using a completely different design setup. Thus, comparing the overall behaviour of a predictive model to the expected behaviour for a completely different dataset is worthwhile.

**Figure 8:**
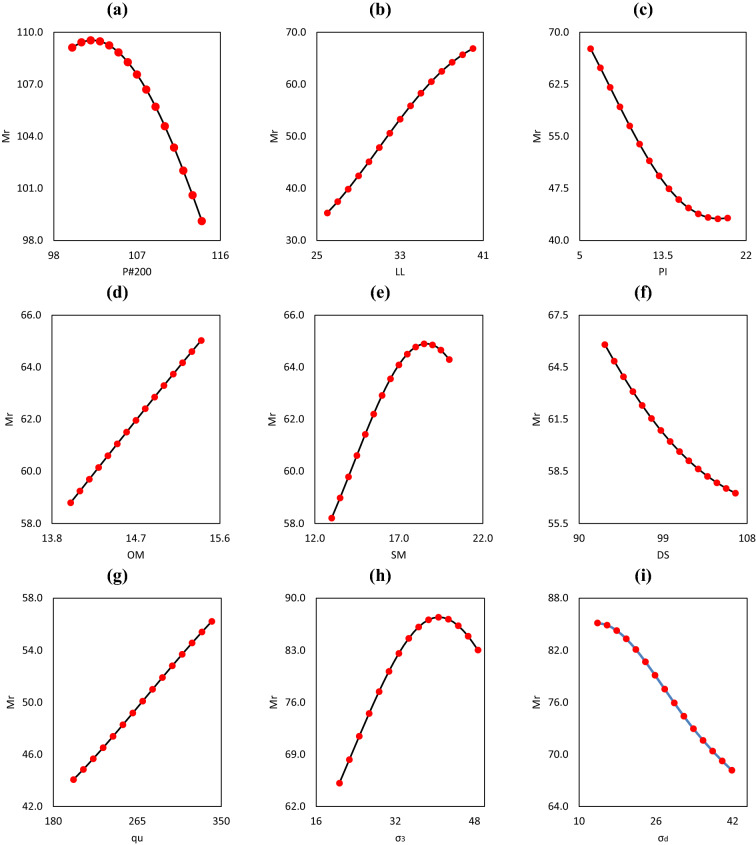
Illustration of behaviour of different input parameters.

In this work, a simulated dataset was constructed to evaluate the robustness, overall behaviour, and expected trend of different input parameters in predicting the Mr of subgrade soils. To generate the simulated datasets, one input parameter was changed while the remaining input parameters remained constant. The details of the simulated datasets are presented in Table 10. Figure 8 shows all of the trends as smooth curves, revealing that when the values of P#200, PI, DS, and σ_d_ increased^63,64^, the Mr of subgrade soils decreased (see Fig. 8a,c,f,i). On the contrary, as the values of LL, OM, SM, q_u_, and σ_3_ increase, the soil Mr increases^63,64^ (see Fig. 8b,d,e,g,h). It is worth noting that the LSSVM-SOS model was used to ensure expected trends of the input parameters using a simulated dataset, but real-life analysis may provide different results. Based on the results of the parametric study, the robustness of the proposed LSSVM-SOS model can be established.

**Table 10:** Details of simulated datasets.

Parameters	Range	Values of constant input parameters	Fig. reference
P#200	100–114	LL = 30; PI = 9; OM = 9.4; SM = 13.1; DS = 68.4; q_u_ = 556.4; σ_3_ = 41.40; σ_d_ = 41.37	Figure 8a
LL	26–40	P#200 = 80; PI = 9; OM = 14.5; SM = 14.5; DS = 84; q_u_ = 325.7; σ_3_ = 41.37; σ_d_ = 67.16	Figure 8b
PI	6.1–20.1	P#200 = 55; LL = 22; OM = 13.8; SM = 13.8; DS = 79.3; q_u_ = 102.7; σ_3_ = 25.8; σ_d_ = 36	Figure 8c
OM	14–15.4	P#200 = 61; LL = 28; PI = 11; SM = 12; DS = 72; q_u_ = 347.6; σ_3_ = 0; σ_d_ = 28.71	Figure 8d
SM	13–20	P#200 = 92; LL = 55; PI = 36; OM = 18.6; DS = 79.8; q_u_ = 282.8; σ_3_ = 0; σ_d_ = 54.46	Figure 8e
DS	92.8–106.8	P#200 = 84; LL = 33; PI = 13; OM = 16; SM = 16; q_u_ = 400; σ_3_ = 41.37; σ_d_ = 68.67	Figure 8f
q_u_	200.4–340.4	P#200 = 100; LL = 59; PI = 32; OM = 24.2; SM = 27.2; DS = 89.2; σ_3_ = 0; σ_d_ = 66.62	Figure 8g
σ_3_	20.69–48.69	P#200 = 62; LL = 26; PI = 9; OM = 13; SM = 11.9; DS = 74.6; q_u_ = 620.5; σ_d_ = 39.67	Figure 8h
σ_d_	13.81–41.81	P#200 = 62; LL = 26; PI = 9; OM = 13; SM = 12.9; DS = 83.3; q_u_ = 535.3; σ_3_ = 41.37	Figure 8i

## Conclusions

In the present study, six swarm intelligence meta-heuristic algorithms (PSO, GWO, SOS, SSA, SMA, and HHO) are applied to optimize the LSSVM parameters for developing a new hybrid technique that can be used for modelling Mr of subgrade soils. 891 datasets of different sites included confining stress, deviator stress, unconfined compressive strength, degree of soil saturation, soil moisture content, plasticity index, percentage of soil particles passing through a #200 sieve, liquid limit, optimum moisture content were used to design and test the proposed models; in addition, the significance of these variables was investigated. The statistical evaluations of the proposed models (i.e., LSSVM-PSO, LSSVM-GWO, LSSVM-SOS, LSSVM-SSA, LSSVM-SMA, and LSSVM-HHO) demonstrate that the LSSVM-GWO, LSSVM-SOS, and LSSVM-SSA outperform other models in the prediction of Mr of subgrade soils. The (RMSE and R^2^) of the LSSVM-GWO, LSSVM-SOS, and LSSVM-SSA are (6.79 MPa and 0.940), (6.72 MPa and 0.942), and (6.78 MPa and 0.940), respectively. The statistical indices visual and uncertainty evaluations conclude that the LSSVM-SOS model performs well in predicting the Mr values and can be used as a superior model in this study. The obtained results for the sensitivity input variables on modelling Mr indicate that all variables positively impact Mr Values. However, the impact of % passing No. 200 sieve, optimum moisture content, and unconfined compressive strength was found more significant in Mr modelling subgrade soils.

The comparison of LSVM-SOS with state-of-the-art models shows that it is extremely efficient in detecting an accurate Mr value for the subgrade soils. However, the future direction of this study may include (a) a comprehensive assessment of the accuracy of the proposed LSSVM-SOS and other hybrid LSSVMs using other datasets of Mr from other regions; (b) an assessment of results of other hybrid models (such as ANNs, ELMs, ANFIS, etc.) constructed with swarm intelligence algorithms^13,65–67^, physics-based algorithms, evolutionary algorithms and human-based OAs; and (c) implementation of different mechanisms such as PSO-based mutation mechanism^67^, adaptive and time-varying acceleration coefficients^68–71^, Gaussian-based mutation with an exploratory search mechanism^72^, etc. to improve the performance of hybrid models constructed with standard version of OAs. Nonetheless, to the authors' knowledge, this is the first study to apply hybrid LSSVM models created with a specific set of OAs (i.e., swarm intelligence algorithms) to estimate the Mr of subgrade soils.

## Data Availability

The data used in this study is available in reference^3^ and online at https://etd.ohiolink.edu/apexprod/rws_olink/r/1501/10?clear=10&p10_accession_num=osu1190140082.

## References

[1] AASHTO. *AASHTO: T307-99 Standard Method of Test for Determining the Resilient Modulus of Soils and Aggregate Materials*. (American Association of State Highway and Transportation Officials, 2003).

[2] AASHTO. Standard method of test for resilient modulus of subgrade soils and untreated base/subbase materials. *Test Procedure T307, AASHTO* (2017) 10.1155/2014/372838.

[3] Hanittinan, W. Resilient modulus prediction using neural network algorithms. *PhD thesis, The Ohio State University* (2007).

[4] Pal M, Deswal S (2014). Extreme learning machine based modeling of resilient modulus of subgrade soils. Geotech. Geol. Eng..

[5] George, K. P. *Prediction of Resilient Modulus from Soil Index Properties*. *Report FHWA/MS-DOT-RD-04-172* (2004).

[6] Kolisoja P (1997). Materials, Resilient Deformation Characteristics of Granular Materials.

[7] Lekarp F, Isacsson U, Dawson A (2000). State of the art. I: Resilient response of unbound aggregates. J. Transp. Eng..

[8] Cary CE, Zapata CE (2011). Resilient modulus for unsaturated unbound materials. Road Mater. Pavement Des..

[9] Azam AM, Cameron DA, Rahman MM (2013). Model for prediction of resilient modulus incorporating matric suction for recycled unbound granular materials. Can. Geotech. J..

[10] Gabr AR (2021). A novel approach for resilient modulus prediction using extreme learning machine-equilibrium optimiser techniques. Int. J. Pavement Eng..

[11] Kaloop MR (2019). Predicting resilient modulus of recycled concrete and clay masonry blends for pavement applications using soft computing techniques. Front. Struct. Civ. Eng..

[12] Kim S-H, Yang J, Jeong J-H (2014). Prediction of subgrade resilient modulus using artificial neural network. KSCE J. Civ. Eng..

[13] Kaloop MR (2019). Particle swarm optimization algorithm-extreme learning machine (PSO-ELM) model for predicting resilient modulus of stabilized aggregate bases. Appl. Sci..

[14] Kaloop MR (2020). A hybrid wavelet-optimally-pruned extreme learning machine model for the estimation of international roughness index of rigid pavements. Int. J. Pavement Eng..

[15] Chen C, Seo H, Jun CH, Zhao Y (2021). Pavement crack detection and classification based on fusion feature of LBP and PCA with SVM. Int. J. Pavement Eng..

[16] Qi C, Tang X (2018). Slope stability prediction using integrated metaheuristic and machine learning approaches: A comparative study. Comput. Ind. Eng..

[17] Zhang P, Yin Z-Y, Jin Y-F, Chan THT, Gao F-P (2021). Intelligent modelling of clay compressibility using hybrid meta-heuristic and machine learning algorithms. Geosci. Front..

[18] Kardani MN, Baghban A, Hamzehie ME, Baghban M (2019). Phase behavior modeling of asphaltene precipitation utilizing RBF-ANN approach. Pet. Sci. Technol..

[19] Zhang W (2021). Application of deep learning algorithms in geotechnical engineering: A short critical review. Artif. Intell. Rev..

[20] Wang X (2020). A hybrid model for prediction in asphalt pavement performance based on support vector machine and grey relation analysis. J. Adv. Transp..

[21] Karballaeezadeh N (2019). Prediction of remaining service life of pavement using an optimized support vector machine (case study of Semnan-Firuzkuh road). Eng. Appl. Comput. Fluid Mech..

[22] Cheng, M. Y. & Prayogo, D. Modeling the permanent deformation behavior of asphalt mixtures using a novel hybrid computational intelligence. in *ISARC 2016 33rd International Symposium on Automation and Robotics in Construction* (2016). 10.22260/isarc2016/0121.

[23] Cheng MY, Prayogo D, Wu YW (2019). A self-tuning least squares support vector machine for estimating the pavement rutting behavior of asphalt mixtures. Soft Comput..

[24] Ismail S, Shabri A, Samsudin R (2011). A hybrid model of self-organizing maps (SOM) and least square support vector machine (LSSVM) for time-series forecasting. Expert Syst. Appl..

[25] Yusuf F, Olayiwola T, Afagwu C (2021). Application of Artificial Intelligence-based predictive methods in Ionic liquid studies: A review. Fluid Phase Equilib..

[26] Abdar M (2021). A review of uncertainty quantification in deep learning: Techniques, applications and challenges. Inf. Fusion.

[27] Wang L (2020). Efficient reliability analysis of earth dam slope stability using extreme gradient boosting method. Acta Geotech..

[28] Zhang W, Zhang R, Wu C, Goh ATC, Wang L (2020). Assessment of basal heave stability for braced excavations in anisotropic clay using extreme gradient boosting and random forest regression. Underground.

[29] Iqbal M, Zhang D, Jalal FE, Faisal Javed M (2021). Computational AI prediction models for residual tensile strength of GFRP bars aged in the alkaline concrete environment. Ocean Eng..

[30] Ghanbari A, Kardani MN, MoazamiGoodarzi A, JanghorbanLariche M, Baghban A (2020). Neural computing approach for estimation of natural gas dew point temperature in glycol dehydration plant. Int. J. Ambient Energy.

[31] Zhang W, Wu C, Li Y, Wang L, Samui P (2021). Assessment of pile drivability using random forest regression and multivariate adaptive regression splines. Georisk Assess. Manag. Risk Eng. Syst. Geohazards.

[32] Tao L, He X, Wang R (2017). A hybrid LSSVM model with empirical mode decomposition and differential evolution for forecasting monthly precipitation. J. Hydrometeorol..

[33] Aziz, M. A. A., Yasin, Z. M. & Zakaria, Z. Prediction of photovoltaic system output using hybrid least square support vector machine. in *2017 7th IEEE International Conference on System Engineering and Technology (ICSET)*, 151–156 (2017). 10.1109/ICSEngT.2017.8123437.

[34] Su Z, Lu H (2021). Short-term wind power prediction based on hybrid variational mode decomposition and least squares support vector machine optimized by improved salp swarm algorithm model. J. Phys. Conf. Ser..

[35] Xue X (2017). Prediction of slope stability based on hybrid PSO and LSSVM. J. Comput. Civ. Eng..

[36] Wang B, Shahzad M, Zhu X, Rehman KU, Uddin S (2020). A non-linear model predictive control based on grey-wolf optimization using least-square support vector machine for product concentration control in l-lysine fermentation. Sensors.

[37] Thampi SM, Piramuthu S, Berretti KLS, Wozniak M, Singh D (2020). Machine Learning and Metaheuristics Algorithms, and Applications.

[38] Sammen SS (2020). Enhanced artificial neural network with Harris Hawks optimization for predicting scour depth downstream of ski-jump spillway. Appl. Sci..

[39] Li S, Chen H, Wang M, Heidari AA, Mirjalili S (2020). Slime mould algorithm: A new method for stochastic optimization. Futur. Gener. Comput. Syst..

[40] Suykens J, Vandewalle J (1999). Least squares support vector machine classifiers. Neural Process. Lett..

[41] Guo T (2019). An improved LSSVM model for intelligent prediction of the daily water level. Energies.

[42] Kardani MN, Baghban A (2017). Utilization of LSSVM strategy to predict water content of sweet natural gas. Pet. Sci. Technol..

[43] Du D, Jia X, Hao C (2016). A new least squares support vector machines ensemble model for aero engine performance parameter chaotic prediction. Math. Probl. Eng..

[44] Mirjalili S, Mirjalili SM, Lewis A (2014). Grey wolf optimizer. Adv. Eng. Softw..

[45] Cheng M-Y, Prayogo D (2014). Symbiotic organisms search: A new metaheuristic optimization algorithm. Comput. Struct..

[46] Kassaymeh S, Abdullah S, Al-Betar MA, Alweshah M (2021). Salp swarm optimizer for modeling the software fault prediction problem. J. King Saud. Univ. Comput. Inf. Sci..

[47] Tan L, Han J, Zhang H (2020). Ultra-short-term wind power prediction by salp swarm algorithm-based optimizing extreme learning machine. IEEE Access.

[48] Mirjalili S (2017). Salp Swarm Algorithm: A bio-inspired optimizer for engineering design problems. Adv. Eng. Softw..

[49] Zubaidi SL (2020). Hybridised artificial neural network model with slime mould algorithm: A novel methodology for prediction of urban stochastic water demand. Water.

[50] Heidari AA (2019). Harris hawks optimization: Algorithm and applications. Futur. Gener. Comput. Syst..

[51] Ray R (2021). Application of soft computing techniques for shallow foundation reliability in geotechnical engineering. Geosci. Front..

[52] Kardani N, Bardhan A, Kim D, Samui P, Zhou A (2021). Modelling the energy performance of residential buildings using advanced computational frameworks based on RVM, GMDH, ANFIS-BBO and ANFIS-IPSO. J. Build. Eng..

[53] Kardani N (2021). A novel technique based on the improved firefly algorithm coupled with extreme learning machine (ELM-IFF) for predicting the thermal conductivity of soil. Eng. Comput..

[54] Kumar M, Bardhan A, Samui P, Hu JW, Kaloop MR (2021). Reliability analysis of pile foundation using soft computing techniques: A comparative study. Processes.

[55] Asteris PG, Skentou AD, Bardhan A, Samui P, Pilakoutas K (2021). Predicting concrete compressive strength using hybrid ensembling of surrogate machine learning models. Cem. Concrete. Res..

[56] Hintze JL, Nelson RD (1998). Violin plots: A box plot-density trace synergism statistical computing and graphics violin plots: A box plot-density trace synergism. Source Am. Stat..

[57] Gholami A, Bonakdari H, Samui P, Mohammadian M, Gharabaghi B (2019). Predicting stable alluvial channel profiles using emotional artificial neural networks. Appl. Soft Comput. J..

[58] Kim, D.-G. *Development of a Constitutive Model for Resilient Modulus of Soils*. (PhD thesis, The Ohio State University, 2004).

[59] Ghorbani B, Arulrajah A, Narsilio G, Horpibulsuk S, Bo MW (2020). Development of genetic-based models for predicting the resilient modulus of cohesive pavement subgrade soils. Soils Found..

[60] Sadrossadat E, Heidaripanah A, Ghorbani B (2018). Towards application of linear genetic programming for indirect estimation of the resilient modulus of pavements subgrade soils. Road Mater. Pavement Des..

[61] Heidarabadizadeh N, Ghanizadeh AR, Behnood A (2021). Prediction of the resilient modulus of non-cohesive subgrade soils and unbound subbase materials using a hybrid support vector machine method and colliding bodies optimization algorithm. Constr. Build. Mater..

[62] Zadeh LA (1975). The concept of a linguistic variable and its application to approximate reasoning I. Inf. Sci..

[63] Sadrossadat E, Heidaripanah A, Osouli S (2016). Prediction of the resilient modulus of flexible pavement subgrade soils using adaptive neuro-fuzzy inference systems. Constr. Build. Mater..

[64] Khasawneh MA, Al-jamal NF (2019). Modeling resilient modulus of fine-grained materials using different statistical techniques. Transp. Geotech..

[65] Hasthi V, Raja MN, Hegde A, Shukla SK (2022). Experimental and intelligent modelling for predicting the amplitude of footing resting on geocell-reinforced soil bed under vibratory load. Transp. Geotech..

[66] Raja MNA, Shukla SK (2021). Predicting the settlement of geosynthetic-reinforced soil foundations using evolutionary artificial intelligence technique. Geotext. Geomembr..

[67] Kardani N, Bardhan A, Roy B, Samui P, Nazem M, Armaghani DJ, Zhou A (2021). A novel improved Harris Hawks optimization algorithm coupled with ELM for predicting permeability of tight carbonates. Eng. Comput..

[68] Ziyu, T., & Dingxue, Z. A modified particle swarm optimization with an adaptive acceleration coefficients. *IEEE, In 2009 Asia-Pacific Conference on Information Processing*, vol. 2, 330–332 (2009).

[69] Cui, Z., Zeng, J., & Yin, Y. An improved PSO with time-varying accelerator coefficients. *IEEE, In 2008 Eighth International Conference on Intelligent Systems Design and Applications*, vol. 2, 638–643 (2008).

[70] Wang B, Zhou M, Xin B, Zhao X, Watada J (2019). Analysis of operation cost and wind curtailment using multi-objective unit commitment with battery energy storage. Energy.

[71] Zhang P, Yin Z-Y, Chen Q (2022). Image-based 3D reconstruction of granular grains via hybrid algorithm and level set with convolution kernel. J. Geotech. Geoenviron. Eng..

[72] Gupta S, Deep K, Mirjalili S (2020). An efficient equilibrium optimizer with mutation strategy for numerical optimization. App. Soft Comp..

